# The characteristics of the suicide attempter according to the onset time of the suicidal ideation

**DOI:** 10.1186/s12991-015-0087-6

**Published:** 2015-12-30

**Authors:** Kotaro Otsuka, Hikaru Nakamura, Kaoru Kudo, Jin Endo, Katsumi Sanjo, Kentaro Fukumoto, Katsuhito Hoshi, Junko Yagi, Akio Sakai

**Affiliations:** Department of Disaster and Community Psychiatry, Iwate Medical University, Morioka, Japan; Department of Neuropsychiatry, Iwate Medical University, Morioka, Japan

**Keywords:** Suicidal attempt, Suicide ideation, Brief psychiatric rating scale (BPRS), Global assessment scale (GAS), Life change units of Holmes social readjustment rating scale (LCU)

## Abstract

**Objective:**

To determine the timing of development of suicidal ideation and factors associated therewith in suicide attempters who required psychiatric emergency treatment.

**Methods:**

Of a total of 2818 suicide attempters in Japan who presented to the primary or secondary emergency department of Iwate Medical University Hospital (hereinafter, referred to as our hospital) or Iwate Prefecture Advanced Emergency and Critical Care Center (hereinafter, referred to as the emergency center), an affiliated institution to our hospital, during the 12-year period from April 1, 2002–March 31, 2014, 2274 patients for whom the timing of development of suicidal ideation was identified were included in the study. The study subjects were classified into three groups according to the timing of development of suicide ideation: the “same-day” group, those who developed suicidal ideation and attempted suicide on the same day; the “short-term” group, those who developed suicidal ideation 2–7 days before attempting suicide; and the “long-term” group, those who developed suicidal ideation more than 7 days before attempting suicide. Factors associated with the development of suicidal ideation in each group were analyzed by a multiple logistic regression analysis with background factors, the diagnosis according to the ICD and the situations before and after the suicide attempt as explanatory variables.

**Results:**

The same-day group was characterized by a high female ratio, high global functioning, low stress level, non-depressed status and a lack of seeking consultation. In contrast, the long-term group was characterized by low global functioning and a high stress level, suggesting that these patients exhibit consultation behavior, but have not received psychiatric services. In the short-term group, only male gender was identified as a significant factor.

**Discussion:**

For those patients who developed suicidal ideation and attempted suicide on the same day, treatment strategies focusing on the acquisition of coping skills and stress management are recommended. For those with suicidal ideation lasting for more than a week or recurrent ideation, early detection and subsequent early treatment of such ideation are essential. In intermediate cases, treatment strategies that make the full use of mental health management in the workplace and gate-keeping are likely to be effective.

## Background

A suicide attempt develops from suicidal ideation in the presence of complicating factors, such as anxiety, impulsivity and underlying psychiatric disorders. Zimri et al. have suggested that the trigger status of suicide is associated with factors such as the intensity of suicidal ideation, history of suicide attempt and current attempts [1], while others emphasize the importance of frantic hopelessness and loss of control over one’s thinking as underlying triggers for suicidal ideation [2]. Some studies have also suggested that psychic pain, a condition characterized by increasing anxiety and restlessness, is experienced before the occurrence of serious suicide attempts [3], and that acute, intense negative feelings can also trigger suicide [4], [5]. However, suicidal ideation is in many cases a distinct entity, and do not always lead to suicide attempts.

Stress has long been identified as an important factor associated with suicide attempt. Mann et al. reported that the severity of current depression or psychosis cannot predict suicide attempt and proposed a stress-diathesis model composed of suicidal ideation, impulsivity, and feelings leading to suicide attempt [6]. While there are various known risk and protective factors associated with suicidal ideation and suicidal behaviors among young people, a population commonly affected by suicide attempts [7], stress has also been shown to have a role in the development of suicidal ideation in this population [8].

Interaction between stress factors and other pertinent factors may complicate stress management and consequently increase the risk of suicide attempt [9]. Controversy remains regarding the notion that stress is a predictor of suicidal tendency [10]. One study suggested that a high stress level does not predict the development of suicidal ideation because the relationship between depression and suicidal ideation is somewhat relaxed by a sudden stress event [11].

Based on the various perspectives, these facts indicate the importance of further analysis of suicidal ideation, which has a close connection to suicide attempt. Regarding the timing of development of suicidal ideation, the prevalence of suicidal ideation within the 2 weeks before suicide attempt is reported to be 4.8 %. Studies have identified the following factors to be associated with the development of suicidal ideation: male gender, high education level, smoking history, living alone, absence of religious behavior, financial strain, history of childhood abuse, family history of suicide, prior history of depression, current anxiety, coexistence of anxiety and depression, prior history of suicide attempt, indifference to one’s own health, and use of antidepressants, as an indirect indicator of current depression, that not necessarily respond to the given drug [12]. To date, no report has fully explored the relationship between the timing of development of suicidal ideation and the characteristics and psychiatric profile of suicide attempters.

The crisis of the true suicide tends to be settled spontaneously in some period. Most patients can adapt themselves to the feelings that have finished becoming impoverished with the risk of the suicide within 24–48 h. The focus of our study is whether psychiatric staffs can management to suicidal ideation on the day and whether they can cope once a week in a generous limit of the short term treatment and what kind of characteristics the patients who no longer maintain suicidal ideation have [13]. Therefore, in this study, we classified suicide attempters who required psychiatric emergency treatment according to the timing of development of suicidal ideation, with the aim to determine the relationship between the time from the development of suicidal ideation to attempted suicide and the nature of suicide attempt.

## Methods

A total of 18,639 patients presented to the primary or secondary emergency department of Iwate Medical University Hospital (hereinafter, referred to as our hospital) or the tertiary emergency department of a hospital-affiliated Advanced Emergency and Critical Care Center (hereinafter, referred to as the emergency center) for psychiatric emergency services during a 12-year period between April 1, 2002 and March 31, 2014.

We use Silverman et al.’s nomenclature for the study of suicidality, and therefore ascribe to their essential components of suicide and suicidal behaviors: suicide ideation, suicide and suicide attempts [14, 15]. Of the total, 2818 patients met the diagnostic criteria for suicide attempt by meeting any of the following criteria: 1) self-statement of suicide attempt, 2) presence of a suicide note or advance notice for suicide, 3) existence of a person witnessing the suicidal behavior, and 4) confirmation by judicial officials or autopsy (Kishi) [16]. Of these 2818 patients, 2274 patients for whom the time of development of suicidal ideation could be identified were included in the analysis (Table 1). Those whose death was confirmed after transportation to the emergency center and those who died within 24 h after admission were defined as suicides

**Table 1:** Timing of development of suicidal ideation

	N	%
Time from development of suicidal ideation to suicide attempt (days)		
Same day	1358	59.7
2–7	492	21.6
>7	424	18.6
Total	2274	100.0

The patients were classified into the following three groups according to the timing of development of suicidal ideation: the “same-day” group, consisting of those who developed suicidal ideation and attempted suicide on the same day; the “short-term” group, consisting of those who developed suicidal ideation 2–7 days before attempting suicide; and the “long-term” group, consisting of those who developed suicidal ideation more than 7 days before attempting suicide. Patients of each group were examined for gender, age, years of schooling, living status, work status, history of presentation to psychiatric services, consultation prior to suicide attempt, history of suicide-related behavior (lifetime and during the past year), motives and means of suicide attempt, Asukai grade [a grading system for the seriousness of attempted suicide based on its means and outcome; absolutely dangerous (AD) or relatively dangerous (RD) group (1995)] [17], Japan Coma Scale (JCS), diagnostic classification according to the Mental and behavioral disorders section of the International classification of diseases and related health problems, 10th edition (hereinafter, referred to as ICD-10) [18], and outcome of emergency treatment. Evaluable patients were also evaluated for psychiatric symptoms using the Oxford University Version of the brief psychiatric rating scale (BPRS) (translated into Japanese by Kitamura et al.) [19] and for overall psychiatric symptoms and daily life capacities using the global assessment scale (GAS) (translated into Japanese by Kitamura et al.) [20]. Because the large part of patients did not be able to diagnose the subgroup for an emergency stage, we investigated the classification of the evaluation to main cord (F1-F9, F99). In addition, the subjects’ life events prior to suicide attempts, such as spouse’s death and debts, were scored on a 0–100 scale using life change units (LCU) of the Holmes social readjustment rating scale [21].

Assessment of each investigation item and diagnoses were based on the information obtained from the patients and those surrounding them, such as family members, paramedics, hospital staff and primary care physicians, at the time of emergency psychiatric services. Suicides were evaluated based on the history of presentation to our department immediately before admission, or in the same manner as described above if they were able to talk at the time of presentation. Investigations and evaluations were conducted by 14 psychiatric emergency psychiatrists or psychiatrists on duty at our hospital, under the supervision of a senior psychiatrist (a designated psychiatrist).

SPSS 21.0 J for Windows was used for statistical processing. One-way analysis of variance was used for comparing the means of three groups (the “same-day”, “short-term”, and “long-term” groups), followed by the analysis of difference between two groups using the Bonferroni method. Chi*-*square test was used for the analysis of ratios among three groups. JCS and LCU data were analyzed using the Kruskal–Wallis test.

In order to identify factors associated with the timing of development of suicidal ideation, a multiple logistic analysis with the forced entry method was performed, with all investigation items (gender, age, years of schooling, living status, work status, diagnosis according to the ICD-10, history of presentation to psychiatric services, history of contact with outpatient psychiatric services, consultation prior to suicide attempt, history of suicide attempt during lifetime and the past year, LCU total score, motives of suicide attempt, first/return presentation, BPRS total score, means of suicide attempt, severity of physical condition (AD/RD group), GAS, and outcome) considered as explanatory variables and the “same-day”, “short-term” and “long-term” groups as the dependent variables.

The significance level was set at 5 % in all tests, with probabilities of significance presented numerically. Personally identifiable data were excluded from the analysis. Data were appropriately managed and processed to ensure the protection of personal information. This study was conducted with the approval of the ethics committees at Iwate Medical University School of Medicine.

## Results

### Background

The same-day group had the largest sample size, followed in order by the short-term and long-term groups. The number of women included was more than twice that of men. The female ratio was especially high in the same-day group compared to the other two groups. The mean age was the highest in the same-day group, followed in order by the short-term and long-term groups. The same-day group had lower percentages of patients who presented for the first time and those who received tertiary emergency services, and higher percentages of patients with prior history of contact with outpatient psychiatric services, history of suicide-related behavior during lifetime or during the past year, and no consultation prior to suicide attempt (Table 2).

**Table 2:** Results of intergroup comparison

Factor	Entire population N = 2274	Time from development of suicidal ideation to suicide attempt			*P* value	Multiple comparison and residual analysis	Analytical method	Unknown N = 544
		Same day N = 1358	Short-term (2–7 days) N = 492	Long-term (>7 days) N = 424				
Male								
N	657	308	188	161	<.001	Shaded area	Chi-square	191
%	28.9	22.7	38.2	38.0				35.1
Age								
Mean	36.73	34.68	38.36	41.43	<.001	Significant difference among 3 groups	Analysis of variance	40.53
SD	16.29	15.11	16.62	18.27				17.46
Presentation to tertiary services								
N	1421	802	331	288	<.001	Shaded area	Chi-square	405
%	62.5	59.1	67.3	67.9				74.4
First-time presentation								
N	1181	629	279	273	<.001	Shaded area		297
%	51.9	46.3	56.7	64.4				54.6
Psychiatric diagnosis								
F0								
N	47	29	12	6	.533			17
%	2.1	2.1	2.4	1.4				3.1
F1								
N	68	49	9	10	.097			12
%	3.0	3.6	1.8	2.4				2.2
F2								
N	310	186	70	54	.802			71
%	13.6	13.7	14.2	12.7				13.1
F3								
N	837	400	210	227	<.001	Shaded area		235
%	36.8	29.5	42.7	53.5				43.2
F4								
N	696	470	132	94	<.001	Shaded area		110
%	30.6	34.6	26.8	22.2				20.2
F6								
N	199	145	39	15	<.001	Shaded area		33
%	8.8	10.7	7.9	3.5				6.1
Other								
N	117	79	20	18	.208			67
%	5.1	5.8	4.1	4.2				12.3
Prior history of suicide attempt								
During the past year								
N	919	611	152	156	<.001	Shaded area		163
%	40.4	45.0	30.9	36.8				30.0
During lifetime								
N	1297	848	239	210	<.001	Shaded area		245
%	57.0	62.4	48.6	49.5				45.0
No consultation prior to suicide attempt								
N	1453	942	297	214	<.001	Shaded area		
%	63.9	69.4	60.4	50.5				
Currently receiving outpatient psychiatric treatment								
N	1411	907	273	231	<.001	Shaded area		
%	62.0	66.8	55.5	54.5				
Means of suicide attempt								
Drug								
N	1221	796	260	165	<.001	Shaded area		276
%	53.7	58.6	52.8	38.9				50.7
Poisoning								
N	137	65	34	38	.004	Shaded area		32
%	6.0	4.8	6.9	9.0				5.9
Inhaling gas								
N	77	27	26	24	<.001	Shaded area		34
%	3.4	2.0	5.3	5.7				6.2
Jumping from a tall building								
N	90	49	16	25	.072			32
%	4.0	3.6	3.3	5.9				5.9
Jumping in front of a train								
N	9	6	1	2	.742			1
%	.4	.4	.2	.5				.2
Knife								
N	399	233	82	84	.385			61
%	17.5	17.2	16.7	19.8				11.2
Self-burning								
N	22	10	7	5	.364			19
%	1.0	.7	1.4	1.2				3.5
Drowning oneself								
N	29	16	4	9	.187			5
%	1.3	1.2	.8	2.1				.9
Hanging								
N	104	49	19	36	<.001	Shaded area		46
%	4.6	3.6	3.9	8.5				8.5
Combination								
N	154	88	38	28	.635			28
%	6.8	6.5	7.7	6.6				5.1
Other								
N	18	10	4	4	.914			2
%	.8	.7	.8	.9				.4
Motives for suicide								
Family problem								
N	502	336	102	64	<.001	Shaded area		108
%	22.1	24.7	20.7	15.1				19.9
Economic difficulties								
N	119	47	40	32	<.001	Shaded area		24
%	5.2	3.5	8.1	7.5				4.4
Pain of sickness								
N	243	112	60	71	<.001	Shaded area		69
%	10.7	8.2	12.2	16.7				12.7
Hallucinations/Delusion								
N	157	82	37	38	.097			17
%	6.9	6.0	7.5	9.0				3.1
Work problem								
N	194	98	54	42	.020	Shaded area		34
%	8.5	7.2	11.0	9.9				6.2
Inter-personal relationship								
N	391	257	87	47	.001	Shaded area		60
%	17.2	18.9	17.7	11.1				11.0
School problem								
N	24	15	3	6	.474			2
%	1.1	1.1	.6	1.4				.4
Other								
N	84	64	13	7	.005	Shaded area		12
%	3.7	4.7	2.6	1.7				2.2
Combination								
N	431	247	86	98	.050			97
%	19.0	18.2	17.5	23.1				17.8
Unknown								
N	129	100	10	19	<.001	Shaded area		121
%	5.7	7.4	2.0	4.5				12.2
Living with someone								
N	1826	1073	400	353	.154			420
%	80.3	79.0	81.3	83.3				77.2
Years of schooling								
Mean	11.70	11.69	11.78	11.64	.663		Analysis of variance	11.63
SD	2.35	2.19	2.39	2.75				2.25
Being at work								
N	786	458	177	151	.110			163
%	34.6	33.7	36.0	35.6				30.0
JCS								
Mean	211.41	196.85	228.89	236.80	.104		Kruskal–Wallis	103.85
SD	373.87	362.48	386.16	392.83				128.43
AD (Asukai classification)								
N	313	129	79	105	<.001	Shaded area	Chi-square	120
%	13.8	9.5	16.1	24.8				22.1
BPRS								
Mean	18.60	17.84	19.10	20.52	<.001	Significant difference between the same-day group and the other groups	Analysis of variance	12.36
SD	11.59	11.44	10.96	12.56				9.80
GAS								
Mean	34.74	37.16	32.99	29.08	<.001	Significant difference among 3 groups		24.81
SD	19.26	19.80	17.77	17.78				18.55
LCU								
Mean	41.70	31.852	29.225	39.378	<.001	Significant difference among 3 groups	Kruskal–Wallis	52.84
SD	33.40	.981	1.476	2.056				37.24
Outcome								
Discharged home								
N	670	471	101	98	<.001	Shaded area		114
%	30.2	35.3	21.0	24.3				22.2
Admitted to emergency care center								
N	592	322	147	123	.004	Shaded area	Chi-square	153
%	26.7	24.1	30.6	30.4				29.8
Admitted to psychiatric department								
N	937	532	225	180	.016	Shaded area		230
%	42.2	39.9	46.9	44.6				44.8
Suicide								
N	36	16	6	14	.007	Shaded area		52
%	1.6	1.2	1.2	3.3				9.6

### Diagnosis, psychiatric parameters, means of suicide attempt and outcome

With respect to the ICD-10 classification, the distribution of mood disorder (F3), neurotic disorder (F4) and personality disorder (F6) significantly varied among three groups, with a higher percentage of F3 and lower percentages of F4 and F6 in the same-day group compared to the other groups.

Regarding the general health performance (GAS average), significant differences were recognized among the three groups, with the long-term group having the highest level of seriousness, followed in order by the short-term and same-day groups. The score for psychiatric symptoms (BPRS total score) was higher in the same-day group compared to the long-term group while the score for life events (LCU average) was the highest in the short-term group, followed in order by the same-day and long-term groups. In terms of motives of suicide attempt, the same-day group had higher percentages of family problems, interpersonal relationship, other, and unknown motive, and a lower percentage of economic difficulties. Higher percentages of work problem and pain of sickness were observed in the short-term and long-term groups, respectively.

Among the means of suicide attempt, drug was the most common in all three groups and its percentage was the highest in the same-day group. The long-term group had higher percentages of poisoning and hanging compared to the other two groups. The percentage of inhaling gas was lower in the same-day group than the other groups. The percentage of patients classified in the AD group according to Asukai’s criteria was the highest in the long-term group (24.8 %).

As for outcome, the percentage of patients discharged home was the highest in same-day group, and the percentages of patients admitted to the emergency center or psychiatric department in the same-day group were higher than the corresponding percentages in the short-term group. There were a total of 36 suicides (1.6 %) and the percentage of suicides was the highest in the long-term group (14 patients, 3.3 %) (Table 2).

### Logistic regression analysis

Significant variables identified in the same-day group included male gender (OR = .682), mood disorder [F3] (OR = .536), consultation prior to suicide attempt (OR = .564), motives of economic difficulties (OR = .415), pain of sickness (OR = .453) and work problem (OR = .391), GAS score (OR = 1.011), and LCU score (OR = .996). In the short-term group, male gender (OR = 1.436) was identified as a significant variable. In the long-term group, first-time presentation (OR = 1.486), consultation prior to suicide attempt (OR = 1925), suicide attempt by drug (OR = .237), GAS score (OR = .986) and LCU (OR = 1.007) were identified as significant variables (Table 3).

**Table 3:** The result of the logistic analysis of each group

Variables in the equation	Same-day group (N = 1358)				Short-term group (N = 492)				Long-term group (N = 424)			
	OR	95 % CI			OR	95 % CI			OR	95 % CI		
		Lower	Upper	P value		Lower	Upper	P value		Lower	Upper	P value
Male (female)	.682	.521	.894	.006	1.436	1.061	1.944	.019	1.154	.833	1.599	.389
Age	.996	.988	1.005	.375	1.000	.990	1.009	.941	1.005	.995	1.016	.311
Tertiary (primary/secondary)	.921	.699	1.213	.557	1.035	.752	1.425	.833	1.090	.769	1.543	.629
First presentation (return)	.816	.634	1.050	.114	.933	.697	1.248	.639	1.486	1.085	2.036	.014
Diagnosis												
F0	.898	.361	2.233	.817	1.906	.707	5.140	.202	.459	.126	1.678	.239
F1	1.598	.674	3.787	.287	.527	.177	1.569	.250	.953	.329	2.756	.929
F2	.766	.405	1.450	.413	1.515	.712	3.224	.281	.896	.396	2.026	.792
F3	.536	.302	.950	.033	1.242	.625	2.467	.536	1.853	.905	3.793	.092
F4	.904	.511	1.601	.729	1.034	.520	2.056	.924	1.071	.517	2.218	.854
F6	.969	.499	1.881	.926	1.641	.757	3.558	.210	.485	.190	1.239	.131
Prior history of suicide attempt												
History of suicide attempt during the past year	1.212	.868	1.691	.259	.593	.404	.870	.008	1.287	.837	1.981	.250
History of suicide attempt during lifetime	1.043	.757	1.437	.799	.982	.689	1.401	.922	.925	.615	1.392	.708
Consultation prior to suicide attempt (no consultation)	.564	.449	.707	.000	1.198	.924	1.552	.172	1.925	1.462	2.535	.000
Prior contact with outpatient psychiatric service (no prior contact)	1.157	.890	1.503	.275	.830	.616	1.120	.223	.995	.722	1.372	.976
Means of suicide attempt												
Drug	3.005	.603	14.970	.179	1.131	.127	10.049	.912	.237	.047	1.206	.083
Poisoning	2.336	.441	12.361	.318	1.141	.121	10.716	.908	.350	.064	1.928	.228
Inhaling gas	1.838	.320	10.552	.495	1.244	.126	12.270	.852	.366	.062	2.147	.266
Jumping from a tall building	2.572	.461	14.356	.281	.684	.067	6.985	.749	.458	.079	2.662	.385
Jumping in front of a train	8.346	.547	127.353	.127	.781	.035	17.325	.876	.000	.000		.999
Knife	2.420	.479	12.233	.285	.924	.102	8.363	.944	.396	.077	2.046	.269
Self-burning	5.057	.728	35.130	.101	.755	.062	9.189	.826	.194	.023	1.620	.130
Drowning oneself	1.340	.196	9.144	.765	.682	.054	8.595	.767	.972	.142	6.644	.977
Hanging	3.025	.547	16.736	.205	.604	.060	6.088	.669	.408	.072	2.322	.313
Combination	1.918	.371	9.926	.437	1.641	.179	15.054	.661	.298	.055	1.605	.159
Other	2.874	.391	21.104	.299	1.161	.090	14.959	.909	.265	.028	2.529	.248
Motives for suicide attempt outcome												
Family problem	.826	.417	1.635	.583	1.221	.552	2.702	.623	1.144	.440	2.977	.782
Economic difficulties	.415	.187	.921	.031	1.659	.678	4.063	.268	2.095	.737	5.952	.165
Pain of sickness	.453	.215	.958	.038	1.471	.621	3.487	.381	2.234	.826	6.039	.113
Hallucinations/Delusion	.580	.265	1.271	.174	1.214	.489	3.015	.676	2.139	.744	6.144	.158
Work problem	.391	.186	.825	.014	1.729	.739	4.046	.207	2.344	.862	6.372	.095
Interpersonal relationship	.571	.285	1.144	.114	1.524	.681	3.411	.305	1.526	.575	4.049	.396
Combined motives	.588	.297	1.163	.127	1.290	.582	2.857	.531	1.844	.718	4.740	.204
School problem	.555	.156	1.977	.363	.718	.132	3.906	.701	3.616	.787	16.613	.098
Unknown motive	1.577	.665	3.741	.301	.418	.136	1.286	.128	1.112	.354	3.495	.856
Living with someone (alone)	.776	.579	1.040	.089	1.265	.898	1.782	.180	1.131	.788	1.623	.505
Years of schooling	.999	.952	1.049	.973	.991	.938	1.048	.756	1.012	.954	1.074	.684
Being at work (not working)	1.074	.837	1.379	.573	.951	.715	1.266	.731	.949	.697	1.291	.738
JCS	1.000	.999	1.000	.378	1.000	1.000	1.000	.935	1.000	1.000	1.001	.375
AD group (RD)	.707	.484	1.033	.073	1.073	.707	1.629	.741	1.406	.918	2.152	.117
Total BPRS	.991	.979	1.002	.104	1.001	.988	1.015	.830	1.012	.999	1.026	.076
GAS	1.011	1.004	1.017	.001	.997	.990	1.004	.450	.986	.978	.994	.001
LCU	.996	.993	1.000	.047	.998	.994	1.002	.363	1.007	1.003	1.011	.001
Outcome												
Discharged home	1.234	.230	6.608	.806	.628	.113	3.480	.594	1.351	.134	13.674	.799
Admitted to emergency center	1.361	.253	7.334	.720	.711	.128	3.943	.696	1.023	.102	10.303	.984
Admitted to psychiatric department	.942	.177	5.014	.944	.843	.154	4.621	.844	1.476	.148	14.774	.740

Variables included: gender, age, primary/secondary/tertiary services, first/return presentation, f0, f1, f2, f3, f4, f6, with or without SMI 1 year for presentation/absence and logistic analysis, with or without history of SMV for logistic analysis, with or without prior consultation for logistic analysis, contact with outpatient psychiatric analysis for logistic analysis, drug, poisoning, inhaling gas, jumping from a tall building, jumping in front of a train, knife, self-burning, drowning oneself, hanging, combination, other, family problem, economic difficulties, pain of sickness, hallucination/delusion, work problem, interpersonal relationship, combined motives, school problem, unknown motive, living with someone or alone, years of schooling, work status, JCS, ADRD, total BPRS, GAS, LCU, discharged home or not, admitted to emergency center or not, admitted to psychiatric department or not

## Discussion

Variables identified by a multivariate analysis as differentiating the three groups were gender, first/return presentation, F3 component of the ICD-10, consultation prior to suicide attempt, drug as a means of suicide attempt, economic difficulties, pain of sickness and work problem as motives for suicide attempt, LCU, and GAS.

### Same-day group

Suicidal ideation that occurred on the day of suicide attempt was significantly associated with female gender, being not depressed, no consultation prior to suicide attempt, suicide attempt by means of drug, suicide attempt not motivated by economic difficulties, pain of sickness or work problem, low LCU, and high GAS. Global function was rated high, suggesting the involvement of vulnerability in coping with stress, with which even life events of low stress levels can lead to suicidal ideation.

Not all suicide-related behaviors are intended for death; some behaviors serve as a subconscious signal for help. Such help-seeking behaviors are particularly notable in women, and are grouped under the concept of “parasuicides” [22, 23]. In the same-day group, it appeared that help-seeking behavior that occurred before suicide attempt did not directly lead to consultation or treatment and did not prevent suicide attempt. Regarding life events, suicide attempts in young people are likely to be triggered by interpersonal relationship-related events, and this risk appears to be higher for women than for men [24, 25]. In order to reduce stress, it is important to maintain good quality of life and integrate problem coping and solving [26]. Ibrahim et al. have suggested that coping skills required for pain management should be reinforced to reduce depression and suicide among young people [26]. In order to prevent the development of suicidal ideation followed by suicide attempt on the same day, supports are needed to facilitate the acquisition of stress management and stress coping skills and the control of mental conditions that can easily lead to direct actions, such as anxiety and impulsivity.

A higher percentage of patients in the same-day group had history of prior suicide attempt and were on outpatient psychiatric treatment compared to the other groups. It should be noted that parasuicide cases who do not have physically serious conditions and have exhibited suicide-related behaviors several times, and those whose suicidal feelings were temporarily weakened after an attempt due to its cathartic effect [27], are very likely to repeat their attempts, finally with a higher rate of fatality [28, 29, 30]. Joiner et al. have identified “acquired suicide potential” as a key risk factor for suicide [31]. This represents an insensitivity to physical pain and lack of fear for suicide acquired through repeated suicide-related behaviors and other similar experiences. These experiences appear to serve as a type of “rehearsal” for suicide and reduce the threshold for suicidal behaviors [32]. Even if a patient is judged to be safe enough to return home after outpatient treatment, it is necessary to determine the process by which he/she came to try to kill him/herself, and to provide careful treatment, such as the introduction of proper psychotherapy or encouragement to visit a psychiatrist in the future. A relatively short interval from the development of suicidal ideation to occurrence of suicide attempt also suggests the need for the proactive use of psychiatric emergency and hot-line call services and providing support to the patient’s family and the surrounding people.

### Short-term group

In the short-term group, only male gender was identified as a significant variable associated with the development of suicide ideation. It is more likely that, compared to women, men do not consult with the people around them prior to suicide attempt and often refuse to see a psychiatrist, even if the people around them notice changes and encourage them to see a psychiatrist. It has also been suggested that men tend to have too much stress themselves without consulting the people around them, and develop psychological tunnel vision [33]. The first thing to be done is to vigorously promote mental health management in the workplace to prevent the development of suicidal ideation and the occurrence of suicide attempt. Given an interval of 1 week from suicidal ideation to attempt, strategies that make the full use of gate-keeping will be effective in preventing these events.

Specifically, it is important to prevent suicide attempts that can occur within a limited time frame by assessing items identified as significant factors in the same-day and long-term groups, such as a history of presentation to psychiatric services, opportunity for consultation prior to suicide attempt, global seriousness, and stressful life events. For example, a treatment strategy consisting of a combination of various types of care has been shown to reduce the severity of depressive symptoms and the development of suicidal ideation among elderly depressed patients [30]. A substantial effect is likely to be achieved by a comprehensive treatment strategy consisting of counseling services that address stress coping, social work services that support problem solving, and drug therapy that includes impulsivity control in its scope.

### Long-term group

In the long-term group, which consisted of those who attempted suicide more than a week after the development of suicidal ideation, significant factors associated with the development of suicidal ideation included first-time presentation, consultation prior to suicide attempt, no use of a drug as a means of suicide attempt, life events associated with a high stress level, and severely impaired global functioning.

This group is characterized by no previous contact with psychiatric services even after consultation with surrounding people. It is well known that suicides tend to have no previous contact with psychiatric services before committing suicidal behavior [34]. In these cases, where serious untreated episodes are likely to be present, the condition itself may direct the patient to use a serious means of suicide other than drugs, or the presence of untreated episodes itself may prevent them from selecting drugs as a means of suicide attempt. Our previous study has demonstrated that lower GAS is associated with an increased frequency of suicide attempts by dangerous means and that GAS predicts the occurrence of dangerous suicide attempt, regardless of gender, age, or even disease entity [35]. Although no correlation to the emergence of AD group was identified in the present study, it should be noted that the selection of a non-drug means of suicide attempt may lead to serious consequences. Although no significant difference was found in the distribution of outcomes among groups, the long-term group had a slightly higher percentage of suicides (3.3 %) compared to the other groups.

Disorders characterized by impulse-control and anxiety may be the most important in predicting the transition from suicidal ideation to suicide attempt [36]. Having not received any treatment indicates that these problems also remain unsolved. Monitoring of these risk factors is essential when conducting risk assessment [37]. Monitoring the impact of early-life and recent events in vulnerable individuals should be part of risk assessment and treatment [37]. In conclusion, for patients with persistent suicidal ideation, the problem-solving approaches should be selected based on a focus on life events associated with high stress levels. These patients should also be introduced to psychiatric treatment through procedures such as screening and gate-keeping.

## Limitations and future research

Although this was a large scale study involving more than 2000 suicide attempters, the investigation was performed at a single institution. The study population also included 544 patients with unknown timing of development of suicidal ideation. This group of patients (the “unknown” group) had a lower female ratio compared to the same-day group, was older compared to the short-term group, and younger than the long-term group. The “unknown” group also had relatively higher percentages of patients with a history of contact with outpatient psychiatric services (63.9 %), and patients with a prior history of suicide attempt during the past year (>40 %) or lifetime (57 %). This group also included a relatively high percentage of patients presenting to tertiary emergency services, including those from whom detailed descriptions of suicidal ideation could not be obtained after awakening from unconsciousness, and those who were not sure of the precise time of onset of suicidal ideation.

## Conclusion

The present study revealed varying characteristics of suicide attempters depending on the timing of onset of suicidal ideation. The group of patients who attempted suicide within 24 h after the development of suicidal ideation was characterized by a higher female ratio, lack of consultation behavior, life events associated with a low stress level, and high global functioning. These patients are likely to be effectively treated by treatment strategies focusing on the acquisition of coping skills and stress management. The long-term group was characterized by life events associated with high stress levels, severely impaired global functioning, and no previous contact with psychiatric services even after consultation with the surrounding people. For these patients with persistent or recurrent suicidal ideation, early control of symptoms and the utilization of social resources with problem-solving intention are necessary. For the short-term group, suicide risk should be reduced by taking into account the factors which were identified as being significantly associated with the development of suicidal ideation in the same-day and long-term groups.

The present results did not provide insight into the relationship between the timing of development of suicidal ideation and outcome. No significant difference was found in terms of diagnostic category, except for diagnoses other than depression in the same-day group, suggesting a greater role of overall psychiatric severity, rather than specific psychiatric conditions, in predicting the outcome of suicide attempts. Future tasks include a more detailed assessment of suicide risk, taking into account factors in addition to the timing of onset of suicidal ideation.

## References

[1] Yaseen ZS, Gilmer E, Modi J, Cohen LJ, Galynke (2012). Emergency room validation of the revised suicide trigger scale (STS-3): a measure of a hypothesized suicide trigger State. PLoS ONE.

[2] Galynker I, Yaseen Z, Briggs J (2014). Assessing risk for imminent suicide. Psychiatr Ann.

[3] Fawcett J, Simon RI, Hales RE (2006). Depressive Disorders. Textbook of suicide assessment and management.

[4] Hendin H, Al Jurdi RK, Houck PR, Hughes S, Turner JB (2010). Role of intense affects in predicting short-term risk for suicidal behavior: a prospective study. J Nerv Ment Dis.

[5] Hendin H, Maltsberger JT, Szanto K (2007). The role of intense affective states in signaling a suicide crisis. J Nerv Ment Dis..

[6] Mann JJ, Waternaux C, Haas GL, Malone KM (1999). Toward a clinical model of suicidal behavior in psychiatric patients. Am J Psychiatry.

[7] Gutierrez PM, Osman A (2008). Adolescent suicide: an integrated approach to the assessment of risk and protective factors.

[8] Overholser JC, Spirito A, Overholser J (2003). Predisposing factors in suicide attempts: life stressors. Evaluating and treating adolescent suicide attempters: from research to practice.

[9] Lee SI, Jung H (2006). Psychosocial risk factors for suicide. Psychiatr Invest.

[10] Zhang XY, Wang HP, Xia Y, Liu XH, Jung EJ (2011). Stress, coping and suicide ideation in Chinese college students. J Adolesc.

[11] Yen S, Shea MT, Pagno M, Sanislow CA, Grilo CM (2003). Axis I and axis II disorders as predictors of prospective suicide attempts: findings from the collaborative longitudinal personality disorders study. J Abnorm Psychol.

[12] Almeida OP, Draper B, Snowdon J, Lautenschlager NT, Pirkis J, Byrne G, Sim M, Stocks N, Flicker L, Pfaff JJ (2012). Factors associated with suicidal thoughts in a large community study of older adults. Br J Psychiatry.

[13] Chiles JA, Strosahl KD (2005). Clinical manual for assessment and treatment of suicidal patients.

[14] Silverman MM, Berman AL, Sanddal ND, O’carroll PW, Joiner TE (2007). Rebuilding the tower of Babel: a revised nomenclature for the study of suicide and suicidal behaviors. Part 1: background, rationale, and methodology. Suicide Life Threat Behav.

[15] Silverman MM, Berman AL, Sanddal ND, O’carroll PW, Joiner TE (2007). Rebuilding the tower of Babel: a revised nomenclature for the study of suicide and suicidal behaviors. Part 2: suicide-related ideations, communications, and behaviors. Suicide Life Threat Behav.

[16] Kishi Y, Hosaka T, Kurosawa H (2000). Proposal for establishment of suicide statistics at emergency care centers. J Clin Exp Med.

[17] Asukai N (1995). Suicide and mental disorders. Psychiatry Clin Neurosci.

[18] World Health Organization (1993). The ICD-10 classification of mental and behavioural disorders: clinical descriptions and diagnostic guidelines—version 1.

[19] Kitamura T, Machizawa S, Maruyama S (1986). Retest reliability of the Oxford University version of the brief psychiatric rating scale (BPRS): a preliminary investigation of a multi-center study sponsored by the National Institute of Mental Health. J Natl Inst Ment Health.

[20] Endicott J, Spitzer RL, Fleiss JL (1976). The global assessment scale: a procedure for measuring overall severity of psychiatric disturbances. Arch Gen Psychiatry.

[21] Holmes TH (1978). Life situations, emotions, and disease. Psychosomatics.

[22] Suzuki H (2000). The realities of attempted suicides at the emergency service of a university hospital. J Clin Exp Med.

[23] Okamoto K, Sakata Y, Shimokawa K (2002). Sex difference in help-seeking behaviors. J J Psychosom Med.

[24] Bridge JA, Goldestein TR, Brent DA (2006). Adolescent suicide and suicide behavior. J Child Psychol Psychiatry.

[25] Hawton K, van Heeringen K (2009). Suicide. Lancet.

[26] Ibrahim N, Amit N, Suen MW (2014). Psychological factors as predictors of suicidal ideation among adolescents in Malaysia. PLoS ONE.

[27] Matsuishi K, Kitamura N, Sato M (2005). Change of suicidal ideation induced by suicide attempt. Psychiatry Clin Neurosci.

[28] Jenkins GR, Hale R, Papanastassiou M (2002). Suicide rate 22 years after parasuicide: cohort study. BMJ.

[29] Suokas J, Suominen K, Isometsä E (2001). Long-term risk factors for suicide mortality after attempted suicide—findings of a 14-year follow-up study. Acta Psychiatr Scand.

[30] Suominen K, Isometsä E, Suokas J (2004). Completed suicide after a suicide attempt: a 37-year follow-up study. Am J Psychiatry.

[31] Joiner TE, Van Orden KA, Witte TK (2009). The interpersonal theory of suicide: guidance for working with suicidal clients.

[32] Schnaideman ES (2005). Suicide as Psychache: a clinical approach to self-destructive behavior.

[33] Bruce ML, Ten Have TR, Reynolds CF, Katz II, Schulberg HC, Mulsant BH (2004). Reducing suicidal ideation and depressive symptoms in depressed older primary care patients: a randomized controlled trial. JAMA.

[34] Vassilas C, Morgan H (1994). Elderly suicides’ contact with their general practitioner before death. Int J Geriatr Psychiatry.

[35] Endo J, Otsuka K, Yoshida T, Nakamura H, Yambe T, Isono H, Chida F (2009). On factors related to risk to life in attempted suicides: comparison between absolutely and relatively dangerous suicides in emergency and critical care center. J J Assoc Emerg Psychiatry.

[36] Borges G, Nock MK, Abad JMH, Hwang I, Sampson NA, Alonso J, Andrade LH, Angermeyer MC, Beautrais A, Bromet E, Bruffaerts R, de Girolamo G, Florescu S, Gureje O, Hu C, Karam EG, Kovess-Masfety V, Lee S, Levinson D, Medina-Mora ME, Ormel J, Posada-Villa J, Sagar R, Tomov T, Uda H, Williams DR, Kessler RC (2010). Twelve Month prevalence of and risk factors for suicide attempts in the WHO World Mental Health Surveys. J Clin Psychiatry.

[37] Pompili M, Innamorati M, Szanto K, Di Vittorio C, Conwell Y, Lester D, Tatarelli R, Girardi P, Amore M (2011). Life events as precipitants of suicide attempts among first-time suicide attempters, repeaters, and non-attempters. Psychiatry Res.

